# Association between body mass index and myopia in the United States population in the National Health and Nutrition Examination Surveys 1999 to 2008: a cross-sectional study

**DOI:** 10.1186/s40001-023-01542-4

**Published:** 2023-12-05

**Authors:** Yaohui Qu, Huamin Huang, Hongxing Zhang

**Affiliations:** 1 First Clinical Medical College, Shandong University of Traditional Chinese Medicine, 16369 Jingshi Road, Jinan, Shandong China; 2 The First Affiliated Hospital of Shandong First Medical University &, Shandong Provincial Qianfoshan Hospital, 16766 Jingshi Road, Jinan, Shandong China

**Keywords:** BMI, Myopia, Linear, Cross-sectional study

## Abstract

**Background:**

This study investigated the association between body mass index (BMI) and myopia in the United States.

**Methods:**

This cross-sectional study included 8,000 participants from the 1999 to 2008 National Health and Nutrition Examination Survey (NHANES). BMI was classified into four groups: < 18.5, 18.5 – 24.9, 25–29.9, and > 29.9. Three diagnostic thresholds were used for myopia A\B\C: spherical equivalent ≤ −0.5\−0.75\−1 diopters in the right eye. Multivariate logistic regression analysis and smooth curve fitting were performed to evaluate the association between BMI and myopia.

**Results:**

The incidence of myopia was 39.4%. BMI was correlated with myopia, with each 1 kg/m^2^ increase in BMI associated with a 1% increase in the risk of myopia (OR, 1.01; 95% CI 1.01 1.02; *p* < 0.05). In myopia B, after adjusting for confounding factors, compared with the reference group (BMI 18.5–24.9), participants with a BMI of 25–29.9 and greater than 29.9 had a 14% and 25% increased risk of myopia, respectively (OR 1.14; 95% CI 1.01 1.29; *p* = 0.037, OR 1.25; 95% CI 1.08 1.44; *p* = 0.003), which was similar to the results for myopic A (OR, 1.15; 95% CI 1.02 1.3; *p* = 0.027, OR 1.19; 95% CI 1.03 1.37; *p* = 0.018) and myopia C (OR 1.15; 95% CI 1.01 1.31; p = 0.035, OR 1.18; 95% CI 1.01 1.37; *p* = 0.032). Moreover, there was a linear relationship between myopia and BMI (p for nonlinearity = 0.767).

**Conclusions:**

Myopia using all three diagnostic thresholds was positively associated with higher BMI. This suggests a potential association between myopia and higher BMI in the American population, warranting further investigations.

**Supplementary Information:**

The online version contains supplementary material available at 10.1186/s40001-023-01542-4.

## Background

Myopia is described as light rays entering the eye parallel to the optic axis and coming into focus in front of the retina when the ocular accommodation is relaxed [1]. The prevalence of myopia in the United States increased from 25% in 1971–1972 to 41.6% in 1999–2004 [2]. Two reports from China show that the prevalence of myopia among adolescents is 63.1% and 84.8%, and the incidence of high myopia reaches 9.4% and 19.3%, respectively [3, 4]. According to the forecast, the prevalence of myopia will reach 49.8% in 2050, and the prevalence of high myopia will reach 9.8% [5]. Not only do myopic patients suffer from decreased vision, but they are also at significant risk of developing complications, such as myopic macular degeneration, retinal detachment, open-angle glaucoma, and cataracts, which can severely reduce the quality of life [6, 7]. Globally, myopia has become a major public health concern.

Both genetic and environmental factors have an impact on the development of myopia [8–10]. Years of education [11] and hours of outdoor activity [12–14] have been shown to have a strong causal association with myopia and inflammation [15, 16] also correlates with myopia. Then, late sleep [17] and high glycaemic load carbohydrate diets [18] may be potentially associated with myopia. According to the carbohydrate–insulin model of obesity, a high glycaemic load carbohydrate diet leads to an increase in adipose tissue and a greater tendency to become obese [19–21]. Obese individuals tend to have a higher body mass index (BMI) compared to non-obese individuals. However, the results of existing studies on the association between BMI and myopia are inconsistent and mostly focused on Asian populations. Some studies have associated high BMI with myopia [22, 23], but others have linked it to low BMI [24] or have not found an association between both [25, 26].

Therefore, participants in the National Health and Nutrition Examination Survey (NHANES) database from 1999 to 2008 were selected to explore the association between BMI and myopia in the United States population.

## Methods

### Study design and participants

The NHANES is a research program designed to assess the health and nutritional status of adults and children in the United States. This survey is conducted annually with a nationally representative sample of approximately 5,000 people. These individuals are located in counties across the country, 15 of which are visited annually. This study was managed by the National Center for Health Statistics of the Centers for Disease Control and Prevention. The study protocol met the requirements of the Declaration of Helsinki and was approved by the institutional review board of the National Center for Health Statistics. Informed consent was obtained from all participants. A more detailed description of the study protocol is provided elsewhere [27].

**Fig. 1:**
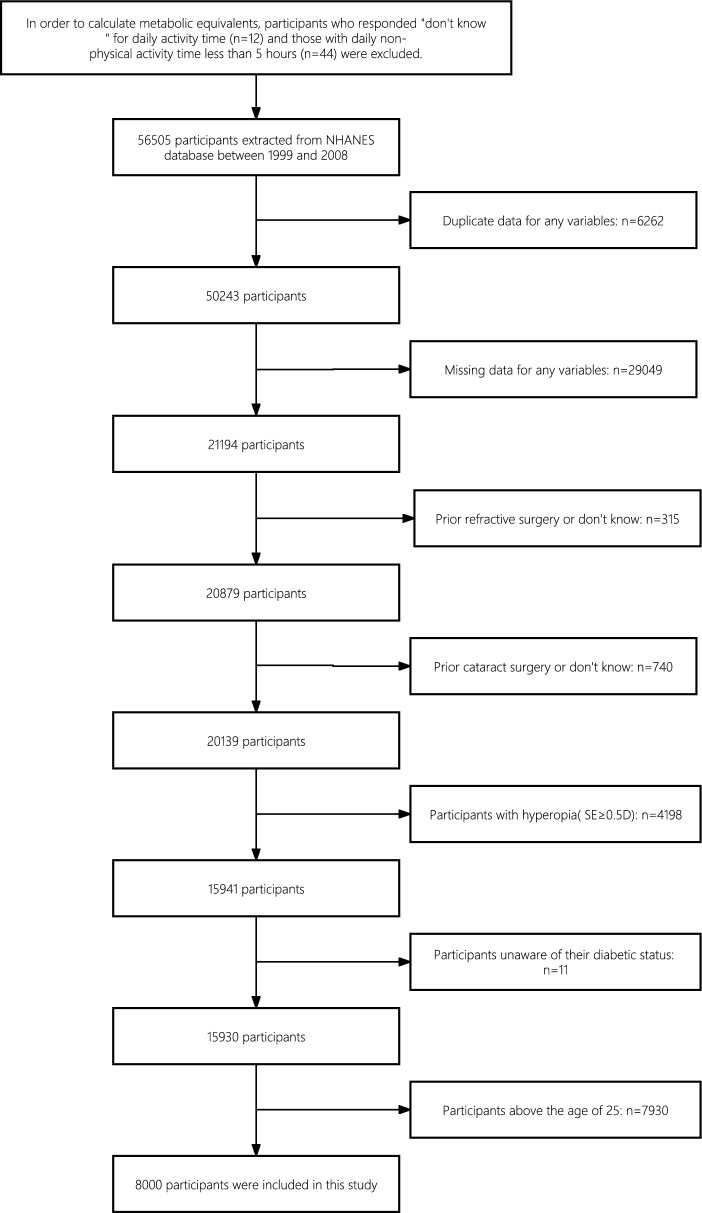
Flowchart detailing the selection process for patients included in this study

This study was a cross-sectional study using data from the NHANES database from 1999 to 2008. To calculate metabolic equivalents, participants who responded "don't know" for daily activity time (*n* = 12) and those with daily non-physical activity time less than 5 h (*n* = 44, because the veracity of the responses was questionable) were excluded. Then, all participants in the survey years 1999–2008 were included (*n* = 56,505). Duplicate data (*n* = 6,262) and missing data (*n* = 29,049) for any variables, and individuals with a history of refractive surgery or do not know (*n* = 315), cataract surgery or do not know (*n* = 740), hyperopia (defined as spherical equivalent ≥ 0.5 diopters (D), *n* = 4,198), unaware of their diabetic status (*n* = 11), above the age of 25 (*n* = 7,930) were excluded. Eventually, 8,000 participants were found suitable for inclusion in our study. The inclusion and exclusion processes are illustrated in Fig. 1.

### Variables and measurement

We included only the right eye as the evaluation eye, because refractive errors in the right and left eyes have been shown to be highly correlated [13]. Technicians who initially received 8 weeks of training and then underwent updates and remedial training as needed performed the visual examination. The objective refraction (sphere and cylinder) results were obtained by taking the average of three measurements using a Nidek Auto Refractor Model ARK-760 instrument. The spherical equivalent was calculated as the sphere plus half the cylinder. Because the NHANES database does not account for cycloplegia in refractive measurements, to ensure reliable results, myopia was diagnosed using three thresholds: myopia A was defined as spherical equivalent ≤ -0.5 D, myopia B was defined as spherical equivalent ≤ −0.75 D and myopia C was defined as spherical equivalent  ≤ -1 D [1, 8, 28, 29].

The professional examiners at the mobile examination center measured the body of each examinee. Information on standing height and weight was collected electronically from the measuring devices to reduce the possibility of data input errors. The US Government Printing Office (https://wwwn.cdc.gov/nchs/nhanes/nhanes3/anthropometricvideos.aspx) offers a specific video technique. BMI was calculated as weight divided by the square of height (BMI = kg/m^2^) and classified into four groups: < 18.5, 18.5–24.9, 25–29.9, and > 29.9 kg/m^2^.

Age, sex (male, female), race (Mexican American, other Hispanic, non-Hispanic White, non-Hispanic Black, and other races), and diabetes data were obtained through personal interviews, and the borderline group for diabetes data was considered to have no diabetes. Physical activity was obtained through the NHANES PAQ questionnaire, and metabolic equivalents were calculated with reference to previous papers [30]. High-density lipoprotein cholesterol (HDL-C) was measured using Hitachi 717 and Hitachi 912 (Roche Diagnostics, 9115 Hague Road, Indianapolis, IN 46250) from 1999 to 2006 and a Roche Modular P chemistry analyzer (Roche Diagnostics, 9115 Hague Road, Indianapolis, IN 46250) from 2007 to 2008. Triglycerides, total cholesterol, glucose, iron, alanine aminotransferase (ALT), and aspartate aminotransferase (AST) levels were measured using Beckman Synchron LX20 and Beckman UniCel^®^ DxC800 Synchron. C-reactive protein (CRP) levels were quantified using latex-enhanced nephelometry.

Missing values were handled by simple deletion, where the proportions of missing values were more than 35% for the following variables: physical activity (44.2%), AST (35.7%), ALT (35.7%), Iron (35.5%), Triglycerides (35.5%), Total cholesterol (35.5%), Glucose (35.5%), cylinder (35.5%). More detailed frequencies and proportions of missing values are shown in Additional file 1: Table S1.

### Statistical methods

For the baseline data of the subjects, the measurement data in accordance with the normal distribution were described by the mean ± standard deviation (SD), the measurement data that did not comply with the normal distribution were described by median (first quartile, third quartile), and the count data were described by *n* (%). A mediation analysis was performed for physical activity and adjusted for age, sex, race, ALT, AST, total cholesterol, triglycerides, HDL-C, glucose, iron, CRP, and diabetes mellitus. Multivariate logistic regression analysis was performed to evaluate the association between BMI and myopia. Model 1 was adjusted for age, sex, physical activity, race, and diabetes mellitus. Model 2 was adjusted for age, sex, physical activity, race, ALT, AST, total cholesterol, triglycerides, and HDL-C. Model 3 was adjusted for age, sex, physical activity, race, ALT, AST, total cholesterol, triglycerides, HDL-C, glucose, iron, CRP, and diabetes mellitus. The smooth curve fitting graph was established and adjusted according to the covariables contained in Model 3. Considering the effect of extreme values, only the middle 95% of BMI data are shown. All analyses were performed using the statistical software package R (http://www.R-project.org, The R Foundation) and Free Statistics software version 1.7 (http://www.clinicalscientists.cn/freestatistics/).

## Results

**Fig. 2:**
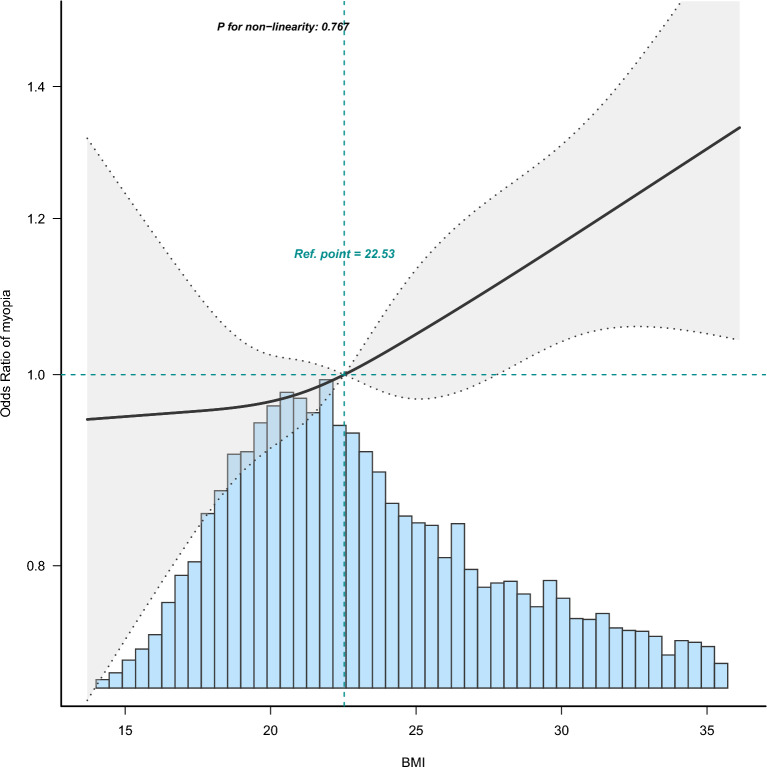
Association between BMI and myopia B odds ratio. Myopia B was defined as spherical equivalent ≤ −0.75 D. Solid and dashed lines represent the predicted value and 95% confidence intervals. They were adjusted for age, sex, physical activity, race, ALT, AST, total cholesterol, triglycerides, HDL-C, glucose, iron, CRP, and diabetes mellitus. Only 95% of the data are shown. *ALT* alanine aminotransferase, *AST* aspartate aminotransferase, *HDL-C* high-density lipoprotein cholesterol, *CRP* C-reactive protein, *BMI* body mass index, *D* diopters

Among 8000 participants with a mean age of 16.9 years, 3149 (39.4%) were diagnosed with myopia B (spherical equivalent ≤ −0.75 D). The baseline characteristics are presented in Table 1. The *p* values for the mediating effect of physical activity on myopia A/B/C were 0.2189/0.184/0.1856, respectively. The results of the multivariate logistic regression analysis of BMI and myopia are presented in Table 2. The trend was the same for all three myopia diagnostic criteria. BMI was correlated with myopia, with each 1 kg/m^2^ increase in BMI associated with a 1% increase in the risk of myopia (OR 1.01; 95% CI 1.01 1.02; *p* < 0.05). In myopia B (spherical equivalent ≤ −0.75 D), compared with the reference group (BMI 18.5–24.9), participants with a BMI of 25–29.9 and greater than 29.9 had a 14% and 25% increased risk of myopia, respectively (OR 1.14; 95% CI 1.01 1.29; *p* = 0.037, OR 1.25; 95% CI 1.08 1.44; p = 0.003), which was similar to the results for myopic A (spherical equivalent ≤ −0.5 D, OR 1.15; 95% CI 1.02 1.3; *p* = 0.027, OR 1.19; 95% CI 1.03 1.37; *p* = 0.018) and myopia C (spherical equivalent ≤ -1 D, OR 1.15; 95% CI 1.01 1.31; *p* = 0.035, OR 1.18; 95% CI 1.01 1.37; *p* = 0.032) in model 3.Smooth curve fitting showed a linear relationship between BMI and myopia B (p for nonlinearity = 0.767, Fig. 2).

**Table 1:** Baseline characteristics of the participants

Variables	Total (*n* = 8000)	BMI (kg/m^2^)				*p* value
		< 18.5 (*n* = 965)	18.5 − 24.9 (*n* = 4203)	25 − 29.9 (*n* = 1558)	> 29.9 (*n* = 1274)	
Sex, *n* (%)						< 0.001
Male	4156 (51.9)	573 (59.4)	2181 (51.9)	829 (53.2)	573 (45)	
Female	3844 (48.0)	392 (40.6)	2022 (48.1)	729 (46.8)	701 (55)	
Age (years)	16.9 (3.3)	14.7 (2.5)	16.8 (3.1)	17.6 (3.4)	18.0 (3.4)	< 0.001
Race, *n* (%)						< 0.001
Mexican American	2559 (32.0)	296 (30.7)	1307 (31.1)	548 (35.2)	408 (32)	
Other Hispanic	464 (5.8)	49 (5.1)	235 (5.6)	99 (6.4)	81 (6.4)	
Non-Hispanic White	2397 (30.0)	307 (31.8)	1307 (31.1)	456 (29.3)	327 (25.7)	
Non-Hispanic Black	2225 (27.8)	251 (26)	1167 (27.8)	399 (25.6)	408 (32)	
Other race	355 (4.4)	62 (6.4)	187 (4.4)	56 (3.6)	50 (3.9)	
Physical activity (METs)	1800.0 (661.5, 4305.0)	1811.2 (721.0, 3840.0)	1939.0 (735.0, 4594.3)	1717.5 (611.6, 4154.5)	1440.0 (481.3, 3840.0)	
Weight (kg)	67.7 (20.0)	44.8 (7.3)	61.0 (9.0)	76.6 (9.8)	99.5 (19.3)	< 0.001
Standing height (cm)	166.1 (10.3)	160.9 (10.7)	166.4 (10.1)	167.5 (9.9)	167.5 (9.9)	< 0.001
ALT (U/L)	17.0 (14.0, 22.0)	15.0 (13.0, 18.0)	16.0 (13.0, 20.0)	19.0 (15.0, 26.0)	22.0 (17.0, 31.0)	< 0.001
AST (U/L)	22.0 (19.0, 26.0)	24.0 (21.0, 28.0)	22.0 (19.0, 26.0)	22.0 (19.0, 27.0)	22.0 (19.0, 27.0)	< 0.001
Total cholesterol (mmol/L)	4.2 (0.9)	4.1 (0.7)	4.1 (0.8)	4.4 (0.9)	4.5 (1.0)	< 0.001
Triglycerides (mmol/L)	0.9 (0.6, 1.2)	0.7 (0.5, 1.0)	0.8 (0.6, 1.1)	1.0 (0.7, 1.4)	1.2(0.8, 1.7)	< 0.001
HDL-C (mmol/L)	1.3 (0.3)	1.5 (0.3)	1.4 (0.3)	1.3 (0.3)	1.2 (0.3)	< 0.001
Glucose (mmol/L)	4.8 (0.9)	4.8 (0.8)	4.8 (1.0)	4.8 (0.8)	5.0 (1.0)	< 0.001
Iron (μmol/L)	14.9 (10.8, 19.7)	15.4 (11.5, 20.1)	15.8 (11.5, 20.6)	14.5 (10.6, 19.2)	12.2(8.6, 16.1)	< 0.001
C-reactive protein (mg/dL)	0.1 (0.0, 0.2)	0.0 (0.0, 0.0)	0.0 (0.0, 0.1)	0.1 (0.0, 0.2)	0.3 (0.1, 0.6)	< 0.001
Diabetes, *n* (%)						< 0.001
Yes	35 (0.4)	3 (0.3)	18 (0.4)	7 (0.4)	7 (0.5)	
No	7965 (99.6)	962 (99.7)	4185 (99.6)	1551 (99.6)	1267 (99.5)	
Myopia A, *n* (%)						< 0.001
Yes	3911 (48.9)	451 (46.7)	1984 (47.2)	796 (51.1)	680 (53.4)	
No	4089 (51.1)	514 (53.3)	2219 (52.8)	762 (48.9)	594 (46.6)	
Myopia B, *n* (%)						< 0.001
Yes	3149 (39.4)	347 (36)	1590 (37.8)	643 (41.3)	569 (44.7)	
No	4851 (60.6)	618 (64)	2613 (62.2)	915 (58.7)	705 (55.3)	
Myopia C, *n* (%)						< 0.001
Yes	2580 (32.2)	287 (29.7)	1299 (30.9)	533 (34.2)	461 (36.2)	
No	5420 (67.8)	678 (70.3)	2904 (69.1)	1025 (65.8)	813 (63.8)	

Categorical variables are reported as No. (%) and continuous variables are reported as mean (SD) or median (Q1, Q3)

Myopia A was defined as spherical equivalent ≤ -0.5 D, myopia B was defined as spherical equivalent ≤ -0.75 D, and myopia C was defined as spherical equivalent ≤ -1 D

*ALT* alanine aminotransferase, *AST* aspartate aminotransferase, *HDL-C* high-density lipoprotein cholesterol, *BMI* body mass index, *MET* metabolic equivalent, *D* diopters, *SD* standard deviation, *Q1* first quartile, *Q3* third quartile

**Table 2:** Association between BMI and myopia in multivariate logistic regression

Variables	Myopia	Crude Model		Adjusted Model 1		Adjusted Model 2		Adjusted Model 3	
		OR (95% CI)	*p* value	OR (95% CI)	*p* value	OR (95% CI)	*p* value	OR (95% CI)	*p* value
BMI (kg/m^2^)	A	1.02 (1.01 ~ 1.02)	< 0.001	1.01 (1.01 ~ 1.02)	< 0.001	1.01 (1.00 ~ 1.02)	0.003	1.01 (1.00 ~ 1.02)	0.012
	B	1.02 (1.01 ~ 1.03)	< 0.001	1.02 (1.01 ~ 1.02)	< 0.001	1.02 (1.01 ~ 1.02)	< 0.001	1.01 (1.01 ~ 1.02)	0.001
	C	1.02 (1.01 ~ 1.02)	< 0.001	1.01 (1.00 ~ 1.02)	0.002	1.01 (1.00 ~ 1.02)	0.009	1.01 (1.00 ~ 1.02)	0.034
< 18.5	A	0.98 (0.85 ~ 1.13)	0.793	1.02 (0.88 ~ 1.17)	0.838	1.02 (0.88 ~ 1.18)	0.771	1.02 (0.88 ~ 1.18)	0.78
	B	0.92 (0.80 ~ 1.07)	0.279	0.96 (0.83 ~ 1.11)	0.594	0.97 (0.83 ~ 1.12)	0.655	0.97 (0.83 ~ 1.12)	0.656
	C	0.95 (0.81 ~ 1.10)	0.479	0.99 (0.85 ~ 1.16)	0.903	0.99 (0.85 ~ 1.16)	0.913	0.99 (0.84 ~ 1.16)	0.902
18.5–24.9	A								
	B	Reference		Reference		Reference		Reference	
	C								
25–29.9	A	1.17 (1.04 ~ 1.31)	0.009	1.16 (1.03 ~ 1.30)	0.014	1.16 (1.02 ~ 1.31)	0.018	1.15 (1.02 ~ 1.30)	0.027
	B	1.15 (1.03 ~ 1.30)	0.017	1.14 (1.02 ~ 1.29)	0.027	1.15 (1.01 ~ 1.30)	0.03	1.14 (1.01 ~ 1.29)	0.037
	C	1.16 (1.03 ~ 1.32)	0.017	1.15 (1.02 ~ 1.30)	0.027	1.16 (1.02 ~ 1.32)	0.024	1.15 (1.01 ~ 1.31)	0.035
> 29.9	A	1.28 (1.13 ~ 1.45)	< 0.001	1.24 (1.09 ~ 1.41)	0.001	1.22 (1.06 ~ 1.40)	0.005	1.19 (1.03 ~ 1.37)	0.018
	B	1.33 (1.17 ~ 1.51)	< 0.001	1.28 (1.13 ~ 1.46)	< 0.001	1.27 (1.10 ~ 1.46)	0.001	1.25 (1.08 ~ 1.44)	0.003
	C	1.27 (1.11 ~ 1.45)	< 0.001	1.22 (1.07 ~ 1.40)	0.004	1.21 (1.05 ~ 1.40)	0.01	1.18 (1.01 ~ 1.37)	0.032

Myopia A was defined as spherical equivalent ≤ -0.5 D, myopia B was defined as spherical equivalent ≤ -0.75 D and myopia C was defined as spherical equivalent ≤ -1 D

Model 1 was adjusted for age, sex, physical activity, race, and diabetes mellitus

Model 2 was adjusted for age, sex, physical activity, race, ALT, AST, total cholesterol, triglycerides, and HDL-C

Model 3 was adjusted for age, sex, physical activity, race, ALT, AST, total cholesterol, triglycerides, HDL-C, glucose, iron, CRP, and diabetes mellitus

*ALT* alanine aminotransferase, *AST* aspartate aminotransferase, *HDL-C* high-density lipoprotein cholesterol, *CRP* C-reaction protein, *BMI* body mass index, *OR* odds ratio, *CI* confidence interval, *D* diopters

## Discussion

In our cross-sectional study that included 8,000 individuals, there was a linear relationship between BMI and myopia (OR 1.01; 95% CI 1.01 1.02; *p* < 0.05). In a multifactorial analysis, participants with a BMI of 25–29.9 and greater than 29.9 had a 14% and 25% increased risk of myopia (spherical equivalent ≤ 0.75 D), respectively (OR 1.14; 95% CI 1.01 1.29; *p* = 0.037, OR 1.25; 95% CI 1.08 1.44; *p* = 0.003), and the trend remained unchanged when the diagnostic threshold was changed to −0.5D (OR 1.15; 95% CI 1.02 1.3; *p* = 0.027, OR 1.19; 95% CI 1.03 1.37; *p* = 0.018)or −1 D(OR 1.15; 95% CI 1.01 1.31; *p* = 0 0.035, OR 1.18; 95% CI 1.01 1.37; *p* = 0.032).The current focus of myopia and BMI research is on Asian children and adolescents. A cross-sectional study that included 1,359,153 Israeli adolescents aged 16–19 years who underwent medical examinations before mandatory military service showed that the BMI was a j-shaped pattern presented in the form of a bar chart for adolescent myopia and both higher and lower BMI were associated with a higher risk of myopia [22]. The results of our study are linear and presented in the form of smooth curve fitting. In the U.S. population, lower BMI did not appear to be associated with myopia, whereas higher BMI similarly had a higher OR. Similarly, a Korean cross-sectional study that investigated 24,269 participants aged 5–18 years in the KNHANES VII database from 2016 to 2018 found an association between obesity and high myopia in childhood and adolescence and an association between overweight and high myopia in girls [23]. According to the results provided in Table 2, there is not enough evidence to show that BMI and myopia are related, which is inconsistent with our results, and the different populations selected may be the source of the difference. A study using the 2003–2008 NHANES database of 6,855 participants aged 12–25 years showed that BMI was not associated with myopia (R2 = 0, *P* = 0.79) [26]. Three possible differences that may have contributed to the different results are as follows: first, the association between BMI and myopia was evaluated using multivariate analysis and adjustment was made with physical activity considered as one of the confounding factors; second, in addition to being analyzed as a continuous variable, BMI was also analyzed in groups. The OR of different groups reached different conclusions. Thirdly, the population from 1999 through 2008 was included, and differences in sample size (8,000) may have contributed to this difference. In a study of 19-year-old male consignors in Seoul, Korea, no association was found between myopia and BMI according to quartiles and by logistic regression [31]. In our study, a higher BMI appeared to favor a risk factor over a protective factor. Differences in population selection may account for the differences in results.

The association between BMI and myopia is affected by underlying lifestyle factors. One review found an association between decreased time spent outdoors and worsening myopia by studying children during COVID-19-induced lockdown [32]. Children who spend more time outdoors may have a lower BMI than their peers [33]. This may be a reasonable explanation for our linear results. After mediating role analysis, it was found that physical activity did not mediate the association between BMI and myopia. It may be one of the confounding factors affecting the results, so we included it in the multivariate analysis to adjust for it.

There are some limitations to this study. First, since the study design was cross-sectional, it was not possible to determine a causal relationship between BMI and myopia. In addition, measurement of refractive error in the absence of cycloplegia increases the prevalence of myopia. Therefore, three mainstream myopia diagnostic criteria were adopted to ensure the reliability of the results. Third, this study did not take into account the impact of air pollution and regional differences as confounding factors and further research is needed to explore this aspect. Fourth, due to the fact that BMI does not consider muscle mass, bone density, overall body composition, or racial and sex differences in lifespan, and also because children’s standards for obesity differ from those for adults, this study can only conclude an association between BMI and myopia, rather than a relationship between obesity and myopia. Fifth, NHANES only provided refractive data from 1999 to 2008, which may be outdated (> 15 years), and newer surveys are still needed to reflect current refractive errors, diet, exercise, or lifestyle.

## Conclusions

In our study, myopia using all three diagnostic thresholds was positively associated with higher BMI. This suggests a potential association between myopia and higher BMI in the American population, warranting further investigations.

### Supplementary Information

**Additional file 1: Table S1.** Frequency and proportion of missing values for all variables.

## Data Availability

Data supporting the findings of this study are available in the NHANES database (https://www.cdc.gov/nchs/nhanes/index.htm). The specific URL for variable acquisition is as follows: 1999–2000. Age, Sex, Race: Demographic Variables & Sample Weights https://wwwn.cdc.gov/nchs/nhanes/search/datapage.aspx?Component=Demographics&CycleBeginYear=1999. Weight, Standing height, BMI: Body Measures (BMX) https://wwwn.cdc.gov/Nchs/Nhanes/Search/DataPage.aspx?Component=Examination&Cycle=1999–2000. ALT, AST, Total cholesterol, Glucose, Iron, Triglycerides: Standard Biochemistry Profile & Hormones (LAB18). https://wwwn.cdc.gov/Nchs/Nhanes/Search/DataPage.aspx?Component=Laboratory&Cycle=1999–2000. “Eye surgery for near sightedness?”, “Eye surgery for cataracts?”, sphere, cylinder: Vision (VIX) https://wwwn.cdc.gov/Nchs/Nhanes/Search/DataPage.aspx?Component=Examination&Cycle=1999–2000. Diabetes: Diabetes (DIQ) https://wwwn.cdc.gov/Nchs/Nhanes/Search/DataPage.aspx?Component=Questionnaire&Cycle=1999–2000. CRP: C-Reactive Protein (CRP) (LAB11) https://wwwn.cdc.gov/Nchs/Nhanes/Search/DataPage.aspx?Component=Laboratory&Cycle=1999–2000. HDL-C: Cholesterol—Total & HDL (Lab13) https://wwwn.cdc.gov/Nchs/Nhanes/Search/DataPage.aspx?Component=Laboratory&Cycle=1999–2000. Physical Activity: Physical Activity – Individual Activities (PAQIAF) https://wwwn.cdc.gov/Nchs/Nhanes/Search/DataPage.aspx?Component=Questionnaire&Cycle=1999–2000 2001–2002. Age, Sex, Race: Demographic Variables & Sample Weights https://wwwn.cdc.gov/nchs/nhanes/search/datapage.aspx?Component=Demographics&CycleBeginYear=2001. Weight, Standing height, BMI: Body Measures (BMX_B) https://wwwn.cdc.gov/Nchs/Nhanes/Search/DataPage.aspx?Component=Examination&Cycle=2001–2002. ALT, AST, Total cholesterol, Glucose, Iron, Triglycerides: Standard Biochemistry Profile (L40_B) https://wwwn.cdc.gov/Nchs/Nhanes/Search/DataPage.aspx?Component=Laboratory&Cycle=2001–2002. “Eye surgery for near sightedness?”, “Eye surgery for cataracts?”, sphere, cylinder: Vision (VIX_B) https://wwwn.cdc.gov/Nchs/Nhanes/Search/DataPage.aspx?Component=Examination&Cycle=2001–2002. Diabetes: Diabetes (DIQ_B) https://wwwn.cdc.gov/Nchs/Nhanes/Search/DataPage.aspx?Component=Questionnaire&Cycle=2001–2002. CRP: C-Reactive protein (CRP), Fibrinogen, Bone Alkaline Phosphatase & Urinary N-telopeptides (L11_B) https://wwwn.cdc.gov/Nchs/Nhanes/Search/DataPage.aspx?Component=Laboratory&Cycle=2001–2002. HDL-C: Cholesterol—Total & HDL (l13_b) https://wwwn.cdc.gov/Nchs/Nhanes/Search/DataPage.aspx?Component=Laboratory&Cycle=2001–2002. Physical Activity: Physical Activity—Individual Activities (PAQIAF_B) https://wwwn.cdc.gov/Nchs/Nhanes/Search/DataPage.aspx?Component=Questionnaire&Cycle=2001–2002 2003–2004. Age, Sex, Race: Demographic Variables & Sample Weights https://wwwn.cdc.gov/nchs/nhanes/search/datapage.aspx?Component=Demographics&CycleBeginYear=2003. Weight, Standing height, BMI: Body Measures (BMX_C) https://wwwn.cdc.gov/Nchs/Nhanes/Search/DataPage.aspx?Component=Examination&Cycle=2003–2004. ALT, AST, Total cholesterol, Glucose, Iron, Triglycerides: Standard Biochemistry Profile (L40_C) https://wwwn.cdc.gov/Nchs/Nhanes/Search/DataPage.aspx?Component=Laboratory&Cycle=2003–2004. “Eye surgery for near sightedness?”, “Eye surgery for cataracts?”, sphere, cylinder: Vision (VIX_C) https://wwwn.cdc.gov/Nchs/Nhanes/Search/DataPage.aspx?Component=Examination&Cycle=2003–2004. Diabetes: Diabetes (DIQ_C) https://wwwn.cdc.gov/Nchs/Nhanes/Search/DataPage.aspx?Component=Questionnaire&Cycle=2003–2004. CRP: C-Reactive Protein (CRP), Bone Alkaline Phosphatase (BAP) & Parathyroid Hormone (PTH) (L11_C) https://wwwn.cdc.gov/Nchs/Nhanes/Search/DataPage.aspx?Component=Laboratory&Cycle=2003–2004. HDL-C: Cholesterol—Total & HDL (l13_c) https://wwwn.cdc.gov/Nchs/Nhanes/Search/DataPage.aspx?Component=Laboratory&Cycle=2003–2004. Physical Activity: Physical Activity—Individual Activities (PAQIAF_C) https://wwwn.cdc.gov/Nchs/Nhanes/Search/DataPage.aspx?Component=Questionnaire&Cycle=2003–2004 2005–2006. Age, Sex, Race: Demographic Variables & Sample Weights https://wwwn.cdc.gov/nchs/nhanes/search/datapage.aspx?Component=Demographics&CycleBeginYear=2005. Weight, Standing height, BMI: Body Measures (BMX_D) https://wwwn.cdc.gov/Nchs/Nhanes/Search/DataPage.aspx?Component=Examination&Cycle=2005–2006. ALT, AST, Total cholesterol, Glucose, Iron, Triglycerides: Standard Biochemistry Profile (BIOPRO_D) https://wwwn.cdc.gov/Nchs/Nhanes/Search/DataPage.aspx?Component=Laboratory&Cycle=2005–2006. “Eye surgery for near sightedness?”, “Eye surgery for cataracts?”, sphere, cylinder: Vision (VIX_D) https://wwwn.cdc.gov/Nchs/Nhanes/Search/DataPage.aspx?Component=Examination&Cycle=2003–2004. Diabetes: Diabetes (DIQ_D) https://wwwn.cdc.gov/Nchs/Nhanes/Search/DataPage.aspx?Component=Questionnaire&Cycle=2005–2006. CRP: C-Reactive Protein (CRP) (CRP_D) https://wwwn.cdc.gov/Nchs/Nhanes/Search/DataPage.aspx?Component=Laboratory&Cycle=2005–2006. HDL-C: Cholesterol—HDL (HDL_D) https://wwwn.cdc.gov/Nchs/Nhanes/Search/DataPage.aspx?Component=Laboratory&Cycle=2005–2006. Physical Activity: Physical Activity—Individual Activities (PAQIAF_D) https://wwwn.cdc.gov/Nchs/Nhanes/Search/DataPage.aspx?Component=Questionnaire&Cycle=2005–2006 2007–2008. Age, Sex, Race: Demographic Variables & Sample Weights https://wwwn.cdc.gov/nchs/nhanes/search/datapage.aspx?Component=Demographics&CycleBeginYear=2007. Weight, Standing height, BMI:Body Measures (BMX_E) https://wwwn.cdc.gov/Nchs/Nhanes/Search/DataPage.aspx?Component=Examination&Cycle=2007–2008. ALT, AST, Total cholesterol, Glucose, Iron, Triglycerides: Standard Biochemistry Profile (BIOPRO_E) https://wwwn.cdc.gov/Nchs/Nhanes/Search/DataPage.aspx?Component=Laboratory&Cycle=2007–2008. “Eye surgery for near sightedness?”, “Eye surgery for cataracts?”, sphere, cylinder: Vision (VIX_E) https://wwwn.cdc.gov/Nchs/Nhanes/Search/DataPage.aspx?Component=Examination&Cycle=2007–2008. Diabetes: Diabetes (DIQ_E) https://wwwn.cdc.gov/Nchs/Nhanes/Search/DataPage.aspx?Component=Questionnaire&Cycle=2007–2008. CRP: C-Reactive Protein (CRP) (CRP_E) https://wwwn.cdc.gov/Nchs/Nhanes/Search/DataPage.aspx?Component=Laboratory&Cycle=2007–2008. HDL-C: Cholesterol—HDL (HDL_E) https://wwwn.cdc.gov/Nchs/Nhanes/Search/DataPage.aspx?Component=Laboratory&Cycle=2007–2008. Physical Activity: Physical Activity (PAQ_E) https://wwwn.cdc.gov/nchs/nhanes/search/datapage.aspx?Component=Questionnaire&CycleBeginYear=2007.

## Abbreviations

D: Diopters
MET: Metabolic equivalent
ALT: Alanine aminotransferase
AST: Aspartate aminotransferase
HDL-C: High-density lipoprotein cholesterol
CRP: C-reaction protein
BMI: Body mass index
SD: Standard deviation
Q1: First quartile
Q3: Third quartile
OR: Odds ratio
CI: Confidence interval
NHANES: National Health and Nutrition Examination Survey
