# Supplementary material for: Association between body mass index and myopia in the United States population in the National Health and Nutrition Examination Surveys 1999 to 2008: a cross-sectional study
Source: Eur J Med Res. 2023 Dec 5;28:561. doi: 10.1186/s40001-023-01542-4 (PMC10696841; doi:10.1186/s40001-023-01542-4)
Supplement: Supplementary file 1 — Additional file 1: Table S1. Frequency and proportion of missing values for all variables. [file 40001_2023_1542_MOESM1_ESM.docx]

Additional Table 1. Frequency and proportion of missing values for all variables.

| Variables | Frequency of missing variables | Percentage of missing variables（%） |
| --- | --- | --- |
| Physical activity (METs) | 22,206 | 44.1972 |
| AST (U/L) | 17,913 | 35.6527 |
| ALT (U/L) | 17,912 | 35.6507 |
| Iron (μmol/L) | 17,833 | 35.4935 |
| Triglycerides (mmol/L) | 17,829 | 35.4855 |
| Total cholesterol (mmol/L) | 17,820 | 35.4676 |
| Glucose (mmol/L) | 17,819 | 35.4656 |
| Cylinder (D) | 17,310 | 34.4526 |
| Sphere (D) | 17,306 | 34.4446 |
| Cataract surgery | 17,171 | 34.1759 |
| Myopia surgery | 17,171 | 34.1759 |
| HDL-C (mmol/L) | 12,012 | 23.9078 |
| C-reactive protein (mg/dL) | 11,107 | 22.1066 |
| BMI (kg/m^2^) | 6,274 | 12.4873 |
| Standing height (cm) | 5,891 | 11.725 |
| Weight (kg) | 3,033 | 6.0367 |
| Diabetes | 1,732 | 3.4472 |
| Age (years) | 1,049 | 2.0879 |
| Race | 2 | 0.004 |
| Sex | 2 | 0.004 |

Abbreviations: MET, metabolic equivalent; ALT, alanine aminotransferase; AST, aspartate aminotransferase; HDL-C, high-density lipoprotein cholesterol; BMI, body mass index; D, diopters.
